# Lightning Strike-Induced Cutaneous Burns and Airway Injury: A Case Report Highlighting Recurrent Extubation Failure in a Critically Ill Patient

**DOI:** 10.3390/ebj7030036

**Published:** 2026-07-01

**Authors:** Robert Canelli, Dhanesh D. Binda, Maxwell B. Baker, Christa M. Lam, Ala Nozari

**Affiliations:** 1 Department of Anesthesiology, Boston Medical Center, Boston University Chobanian & Avedisian School of Medicine, Boston, MA 02118, USA; robert.canelli@bmc.org (R.C.); maxwell.baker@med.uvm.edu (M.B.B.); chlam@bu.edu (C.M.L.); ala.nozari@bmc.org (A.N.); 2 Department of Anesthesiology, Montefiore Medical Center, Albert Einstein College of Medicine, Bronx, NY 10467, USA; 3 Larner College of Medicine, University of Vermont, Burlington, VT 05405, USA

**Keywords:** lightning strike, cutaneous burns, airway burns, extubation failure, Lichtenberg figures, inhalation injury, tracheostomy, intensive care

## Abstract

**Highlights:**

**What are the main findings?**

- Lightning strikes may cause direct oral, supraglottic, and glottic injury, resulting in persistent airway edema, dysphonia, and recurrent extubation failure even in the absence of classic smoke inhalation injury.
- Recurrent extubation failure in our patient was likely multifactorial, involving upper airway injury, pleural effusions, hospital-acquired pneumonia, secretion burden, and neurological impairment, ultimately requiring tracheostomy.

**What are the implications of the main findings?**

- Lightning strike victims with cervical burns or suspected airway involvement should undergo systematic airway evaluation, including a cuff-leak test and upper airway endoscopy before planned extubation.
- Clinicians should maintain a high index of suspicion for occult upper airway injury after lightning strikes, as respiratory tract involvement may occur even without conventional inhalation injury mechanisms.
- Early elective tracheostomy should be considered when multiple extubation risk factors coexist to avoid repeated extubation failures and cumulative airway trauma.

**Abstract:**

Introduction: Lightning strikes cause a unique spectrum of cutaneous burn injuries that differ substantially from conventional thermal or high-voltage electrical burns. Despite the well-documented systemic lethality of lightning injuries, the burn-specific sequelae—including airway involvement and its contribution to ventilatory failure—remain underreported in the burn literature. Case Report: We present a 31-year-old woman who sustained multiple cutaneous burns following a lightning strike, including a circumferential necklace-pattern burn to the neck, a large midline torso burn, and burns to the right lower extremity and foot. Following resuscitation from ventricular fibrillation cardiac arrest, she required mechanical ventilation and experienced three sequential extubation failures before ultimately requiring tracheostomy. We propose that direct supraglottic and glottic injury, together with airway mucosal edema from localized thermal and electrical injury of the neck, compounded further by systemic inflammatory and infectious complications, contributed to her inability to sustain independent ventilation. Conclusions: In lightning strike victims with burns involving the neck or thorax, direct upper airway injury should be actively considered, excluded when possible, and closely monitored as a potential cause of extubation failure. A low threshold for early bronchoscopic airway assessment and conservative extubation protocols is warranted.

## 1. Introduction

Lightning strikes are one of the most powerful natural phenomena capable of inflicting human injury, delivering currents exceeding one million volts over an interval of 0.001 to 0.1 s. In the United States, lightning is responsible for an estimated 400 injuries and 40 deaths annually, though the true incidence is likely underreported [1]. The mortality rate following a lightning strike ranges from 10–30%, and approximately 76% of survivors experience long-term sequelae [2].

The pathophysiology of lightning-related burns differs markedly from that of conventional high-voltage electrical injuries. Because the contact duration is so brief, lightning energy preferentially travels over the body surface via the “flashover” effect rather than penetrating deeply through tissues [3]. As a result, lightning burns are typically superficial and may cover large surface areas, taking characteristic forms including linear burns from vaporized sweat, punctate burns at current exit points, contact burns from heated metallic objects, and—most pathognomically—Lichtenberg figures (fernlike erythematous skin patterns) [2,4].

Despite their predominantly superficial character, lightning burns in anatomically critical locations, particularly the neck and anterior thorax, pose distinct risks. Burns in the cervical region may involve the upper airway mucosa, even in the absence of enclosed-space smoke inhalation, the traditional trigger for suspecting inhalation injury. The possibility that direct thermal injury from a necklace-pattern cervical burn could extend to the supraglottic and glottic mucosa and thereby impair airway patency after extubation has not been systematically examined in the burn literature [5].

We report the case of a lightning strike survivor who presented with extensive cutaneous burns in a pattern consistent with the flashover mechanism. Her protracted course in the Intensive Care Unit (ICU) was marked by three extubation failures before tracheostomy was required. We argue that subclinical airway mucosal involvement, in the context of a circumferential neck burn, was a critical and underrecognized contributor to her ventilatory failure, and we discuss the implications for burn unit airway management protocols.

## 2. Case Report

### 2.1. Patient Demographics and Presentation

A 31-year-old woman with no significant past medical history was transported to the emergency department by emergency medical services after bystander-initiated cardiopulmonary resuscitation (CPR) and subsequent return of spontaneous circulation (ROSC) following ventricular fibrillation cardiac arrest caused by a direct lightning strike. She had been walking her dog on a beach when she was struck and lost consciousness. Emergency medical services defibrillated the patient and administered three doses of epinephrine (1 mg each) per Advanced Cardiovascular Life Support (ACLS) guidelines. She was intubated in the field and arrived with oxygen saturations in the 70% range and a Glasgow Coma Scale score of 3T.

### 2.2. Burn Description and Distribution

**Figure 1:**
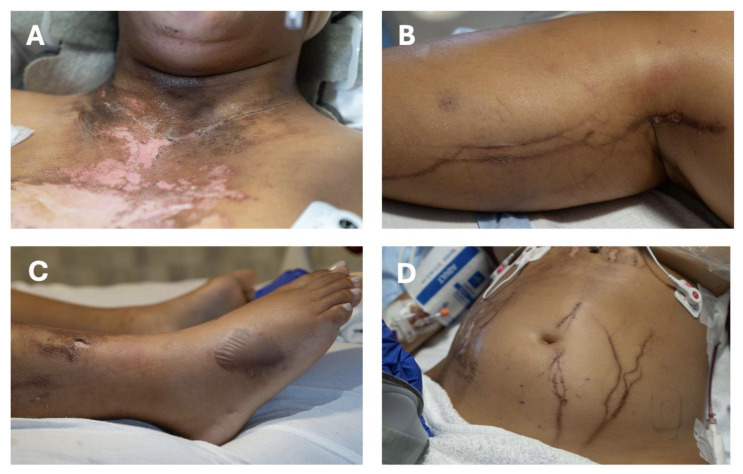
Pathway of lightning strike evidenced by dermal injuries and material damage on a patient with darker skin complexion. (**A**) Initial strike on chest and necklace burn. (**B**) Path of electricity across right popliteal fossa to right calf. (**C**) Burn on right lateral foot demonstrating location of electricity exit from patient. (**D**) Path of grounding across abdomen.

Secondary survey on arrival to the emergency department identified four distinct burn regions consistent with the flashover mechanism of lightning injury (Figure 1). The most clinically significant finding from a burn standpoint was a circumferential necklace-pattern burn encircling the entire neck (Figure 1A). This linear burn followed the anatomical path of the patient’s necklace, which had been heated by the lightning current, producing a full-thickness contact burn in direct proximity to the supraglottic airway.

Burns to the right posterior leg, spanning the popliteal fossa and calf, represented a secondary arc pathway (Figure 1B). A punctate burn on the right lateral foot marked the current exit point (Figure 1C). A large midline torso burn extending from the upper chest across the abdomen was also identified, consistent with a flashover arc following the anterior surface of the body (Figure 1D). A left parietal scalp laceration was also documented. Collectively, these injuries were consistent with lightning entry via the head and scalp, propagation of current over the anterior torso and around the neck, with exit through the right lower extremity.

Initial wound care consisted of bacitracin ointment applied to all burn surfaces; silver sulfadiazine was added several days later. The patient was monitored closely for compartment syndrome throughout her ICU course, and serum creatine kinase (CK) was measured every six hours. No escharotomy was required.

### 2.3. Airway Management and Extubation Failures

The patient arrived intubated with an endotracheal tube initially found in the right mainstem bronchus on chest radiograph. Oxygen saturations fell to 60%, and the tube was repositioned above the carina with immediate improvement. Initial ventilator settings were: FiO_2_ 35%, respiratory rate 16 breaths per minute, tidal volume 350 mL, and PEEP 5 cmH_2_O. On hospital day 4, the patient developed worsening hypoxemia with increasing oxygen requirements, requiring FiO_2_ up to 80%. Chest radiography and bedside ultrasonography demonstrated bilateral pleural effusions, more pronounced on the left, along with bilateral pulmonary infiltrates consistent with moderate acute respiratory distress syndrome. A left-sided 12-Fr chest tube was placed under ultrasound guidance, resulting in immediate drainage of approximately 200–250 mL of serosanguinous fluid. Intravenous fluids were discontinued, diuresis with furosemide was initiated, and lung-protective ventilation strategies were continued. No right-sided pleural drainage procedure was required. By hospital day 10, the patient tolerated minimal pressure support ventilation, at which point extubation was attempted.

**Figure 2:**
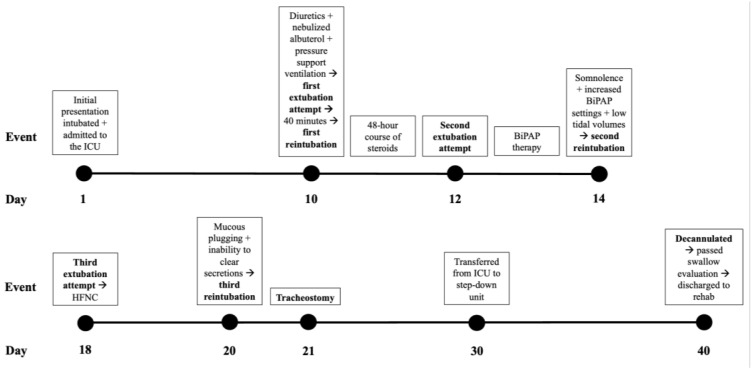
Clinical timeline of airway management and extubation outcomes following lightning strike injury. The patient underwent three extubation attempts on hospital days 10, 12, and 18, each resulting in respiratory compromise and reintubation. Following the third extubation failure, tracheostomy was performed on hospital day 21. The patient was subsequently transferred out of the ICU, successfully decannulated, passed a swallowing evaluation, and was discharged to acute rehabilitation on hospital day 40. ICU, intensive care unit; BiPAP, bilevel positive airway pressure; HFNC, high-flow nasal cannula.

Prior to the first extubation attempt, diuretics and nebulized albuterol were administered (Figure 2). Despite these precautions, the patient maintained independent breathing for only 40 min before developing progressive tachypnea with oxygen saturations falling to the low 70s. She reported subjective dyspnea and fatigue and requested reintubation, which was performed with a smaller-calibre endotracheal tube. The clinical suspicion at this point was airway edema related to the circumferential neck burn, and a 48-h course of systemic corticosteroids was administered before a second extubation attempt on hospital day 12.

This second extubation likewise failed, with tachypnea and oxygen saturations in the mid-80s. Bilevel positive airway pressure (BiPAP) temporarily stabilized oxygenation. However, within two days, the patient became increasingly somnolent on 100% FiO_2_, with tidal volumes falling to 100–150 mL, necessitating a second reintubation. A third extubation attempt was subsequently performed on hospital day 18, and the patient was transitioned to high-flow nasal cannula. However, within 48 h, she developed mucus plugging and an inability to adequately clear secretions, resulting in recurrent respiratory compromise and a third reintubation. Given three sequential extubation failures with a pattern of progressive upper airway and ventilatory compromise, a decision was made to proceed with elective tracheostomy on hospital day 21.

The patient tolerated the tracheostomy without complication. Initial suctioning requirements were hourly, suggesting a significant ongoing airway secretion burden. After transition to a cuffless tracheostomy tube, suctioning frequency decreased to every eight hours, and she was fitted with a speaking valve. She was transferred from the ICU to a step-down unit after four weeks, and by hospital day 40, was successfully decannulated, passed a swallowing evaluation, and discharged to an acute rehabilitation facility. She remained free of tracheostomy dependence throughout follow-up. At 2 months after discharge, her voice was mildly raspy, and continued speech therapy was recommended. Her dysphonia gradually resolved, with no further documentation of voice impairment by 4 months after discharge.

### 2.4. Additional Diagnostic Findings

Initial laboratory investigations revealed leukocytosis of 46 K/μL (attributed to a leukemoid reaction), peak troponin of 15,563 ng/L (decreasing to 1817 ng/L on day 2), and peak creatine kinase of 3169 U/L, consistent with rhabdomyolysis. CT imaging of the chest, abdomen, and pelvis demonstrated diffuse multifocal ground-glass opacities (pulmonary edema/contusions). CT brain demonstrated global cerebral edema. MRI brain identified small infarcts in the left caudate head and bilateral cerebellar hemispheres. Bedside transthoracic echocardiography demonstrated global hypokinesis with an ejection fraction of 50%. No significant changes in ventricular function were observed on subsequent cardiac evaluation. Initial electrocardiography (ECG) showed sinus tachycardia, rightward axis deviation, and a right bundle branch block, which resolved on a repeat ECG obtained approximately 90 min later. A chest CT two weeks after admission revealed left lower lobe consolidation consistent with pneumonia, with respiratory cultures growing Staphylococcus aureus; antibiotics were adjusted accordingly.

Bedside flexible bronchoscopy performed during the ICU stay demonstrated minimal secretions with Gram-positive colonies, but no formal structured assessment of the supraglottic or glottic mucosa for edema, erythema, or friability was documented at the time of the extubation failures.

## 3. Discussion

### 3.1. The Spectrum of Lightning-Induced Cutaneous Burns

Lightning burns occupy a distinct niche in the burn literature. Unlike conventional thermal burns or high-voltage electrical injuries from power lines, lightning burns rarely cause deep tissue destruction because the brevity of current contact precludes sufficient energy transfer for extensive electrothermal heating [3]. The predominant mechanism is the flashover effect, in which current arcs over the moist skin surface, causing superficial injury over wide anatomical territories [6]. This case illustrates the classical anatomical distribution of lightning burns: linear neck burn following a metallic necklace, broad anterior torso flashover burn, posterior leg arc burn, and a punctate exit burn on the lateral foot.

Lichtenberg figures were not documented in this patient, possibly because their transient appearance may be more difficult to recognize in patients with Fitzpatrick skin types IV–VI [7]. Notably, published reports of Lichtenberg figures have been described almost exclusively in patients with lighter skin tones. Our case, therefore, highlights an important gap in the literature and underscores the need to improve recognition of lightning-related dermatologic findings across diverse skin tones to support equitable clinical care [4].

Contact burns produced by heated metallic jewelry can be full-thickness and sharply demarcated. When these occur at the neck, the burn encompasses the anterior and lateral cervical soft tissues in proximity to the larynx and upper trachea. Lightning current itself may cause direct electrical and thermal injury to the supraglottic and glottic mucosa independent of overlying skin burns. Cervical soft tissue burns may further potentiate this injury through local inflammatory mediator release and edema [8].

### 3.2. Airway Burns and the Mechanism of Recurrent Extubation Failure

Inhalation injury in conventional burn patients is a well-established predictor of extubation failure and prolonged mechanical ventilation [8,9]. The triad of airway mucosal edema, impaired mucociliary clearance, and bronchospasm leads to increased work of breathing, atelectasis, and susceptibility to pneumonia. In lightning strike victims, this mechanism has not been systematically described since lightning injuries rarely occur in the context of smoke or superheated gas inhalation.

We propose that a functionally analogous process may occur in patients with cervical lightning burns, even in the absence of classic inhalation injury. The circumferential cervical distribution of injury adjacent to a metallic necklace suggests a localized concentration of electrical current and heat dissipation, potentially amplifying soft tissue and airway damage. In this case, thermal injury to the superficial cervical tissues may have produced sufficient edema and mucosal fragility to critically narrow the upper airway after endotracheal support was withdrawn. This hypothesis is supported by the clinical course. Each extubation failure was characterized by rapid-onset tachypnea and hypoxia, consistent with increased resistive work of breathing from upper airway narrowing rather than intrinsic lung parenchymal failure. The transient benefit of BiPAP after the second failure also supports a component of dynamic upper airway collapse, partially offset by positive airway pressure. The decision to administer systemic corticosteroids after the first failure, a strategy used in conventional inhalation injury to reduce airway edema, lends further implicit credence to the hypothesis that mucosal swelling was one of the significant drivers.

It is also important to acknowledge that lightning current may independently cause direct supraglottic and glottic mucosal injury without overlying cutaneous burns [5,10]. In our patient, direct mucosal injury from the lightning strike may have been a primary contributor to both the recurrent extubation failures and the prolonged dysphonia. Since formal upper airway endoscopy was not performed at the time of the extubation failures, the precise extent and location of injury could not be determined. However, the combination of a circumferential cervical burn, repeated airway compromise after extubation, and dysphonia that persisted for several months supports the possibility of clinically significant supraglottic or glottic injury.

**Figure 3:**
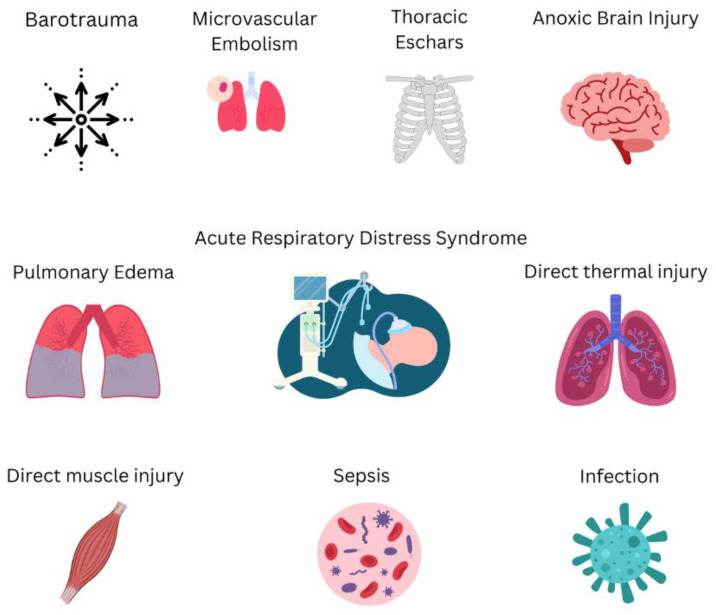
Proposed hypothetical multifactorial mechanism contributing to recurrent extubation failure following lightning strike injury. Circumferential cervical burn from lightning-heated metallic jewelry likely caused supraglottic and upper airway mucosal edema through direct thermal injury and inflammatory soft-tissue swelling. Additional contributors included pulmonary edema and pleural effusions after cardiac arrest and aggressive resuscitation, hospital-acquired pneumonia with secretion burden, and neurological impairment from cerebral edema and ischemic infarcts, collectively increasing respiratory workload and impairing airway protection.

Compounding the airway mucosal injury were several co-existing factors that increased the cumulative respiratory burden: bilateral pleural effusions with a large left-sided effusion requiring chest tube drainage, hospital-acquired staphylococcal pneumonia with left lower lobe consolidation, high secretion burden, and neurological impairment from global cerebral edema and scattered infarcts that likely depressed central respiratory drive and protective airway reflexes (Figure 3). Secretion burden and neurological status are both incorporated into validated extubation and decannulation tools, including the Murray Secretion Scale [10] and the ExPreS and VISAGE scores [11,12], underscoring their relevance to extubation vulnerability in complex critically ill patients. Restrictive chest wall mechanics from the midline torso burn also warrant consideration, as circumferential or near-circumferential thoracic eschars can impede chest wall and diaphragmatic excursion [13]. Although our patient’s torso burn was not circumferential and escharotomy was not performed, reduced chest wall compliance cannot be entirely excluded as a contributing factor.

### 3.3. Absence of Bronchoscopic Upper Airway Assessment

A notable limitation in this case was the absence of formal laryngoscopic or bronchoscopic assessment of the upper airway before or after extubation attempts. Although bronchoscopy was performed during the ICU stay, it documented only distal airway findings and did not include a structured evaluation of the supraglottic or glottic mucosa. We suggest that lightning strike victims with cervical burns or suspected upper airway involvement undergo a cuff-leak test and formal upper airway endoscopic evaluation before planned extubation [14]. The cuff-leak test provides a simple bedside surrogate for subglottic and glottic edema and can help identify patients at risk for post-extubation stridor [15]. A low or absent leak should prompt further airway evaluation, corticosteroid therapy, and reassessment before extubation.

### 3.4. Burn Wound Management Considerations

Initial management with bacitracin ointment followed by silver sulfadiazine was appropriate for the acute ICU setting where systemic priorities dominate. Silver sulfadiazine provides broad-spectrum antimicrobial coverage widely used in burn units for partial- and full-thickness burns [16]. The circumferential nature of the neck burn raises the theoretical concern for progressive swelling and extrinsic airway compression. In practice, the patient was already intubated throughout the acute burn phase, affording airway protection during the period of maximal swelling (typically 24–72 h post-injury). However, the persistence of airway edema beyond this window, evidenced by extubation failures at day 10 and prolonged dysphonia after decannulation, suggests that mucosal injury and edema may have been more important contributors than extrinsic soft-tissue swelling. Although not directly visualized, an oral, supraglottic, and/or glottic lightning-induced burn remains a plausible explanation for these findings.

Escharotomy, when indicated for circumferential or restrictive truncal burns, remains an essential component of burn wound management. Eschar-related chest wall restriction can independently impair ventilation and delay extubation, with timely escharotomy reported to facilitate rapid ventilatory improvement [13]. Lightning-induced rhabdomyolysis, with a peak CK of 3169 U/L in this case, carries a risk of acute tubular necrosis requiring aggressive fluid resuscitation, which may in turn exacerbate pulmonary and airway mucosal edema [17]. High-voltage electrical injuries have also been associated with profound transient hypokalemia through catecholamine-mediated intracellular potassium shifts and disruption of voltage-gated potassium channels. This underrecognized metabolic complication warrants close electrolyte monitoring in the acute setting [18].

### 3.5. Implications for Burn Unit Practice

Our case offers several practical lessons for burn clinicians. First, airway vigilance should be guided not only by the location of external burns but also by the possibility of direct upper airway injury from the lightning current itself. Such injury may occur even in the absence of conventional inhalation injury and should be actively excluded early in the clinical course. Second, extubation readiness assessments should incorporate a cuff-leak test and, where possible, direct laryngoscopy. Third, the risk of recurrent extubation failure is elevated when cervical burns co-exist with systemic factors, and clinicians should have a low threshold for elective tracheostomy rather than repeated intubation–extubation cycles. Fourth, the high suctioning requirements after tracheostomy in this patient suggest that “minimal” distal airway secretions on bronchoscopy may underestimate the true upper airway secretion burden.

## 4. Conclusions

Lightning strike-induced burns present a distinctive pattern of cutaneous injury that differs fundamentally from conventional thermal or high-voltage electrical burns. In this case, a circumferential neck burn produced by metallic jewelry heated by the lightning current was anatomically situated to generate thermal injury to the adjacent supraglottic airway, contributing, alongside pulmonary and neurological complications, to three sequential extubation failures and the eventual need for tracheostomy.

The key take-home messages for burn clinicians are: (1) cervical lightning burns should trigger the same systematic airway evaluation applied to inhalation injury victims; (2) a cuff-leak test and direct upper airway endoscopy should be performed before planned extubation in this population; (3) systemic corticosteroids may attenuate mucosal edema in the peri-extubation period but are insufficient when compounding factors are present; and (4) early elective tracheostomy should be considered when multiple extubation risk factors coexist, to prevent cumulative laryngeal injury and to facilitate airway clearance, communication, and rehabilitation.

Further prospective studies of airway outcomes in lightning burn victims with cervical injury are needed to define optimal surveillance and extubation protocols for this uncommon but high-stakes patient population.

## Data Availability

The data presented in this study are available on request from the corresponding author due to privacy restriction.

## Abbreviations

The following abbreviations are used in this manuscript:

CPR	Cardiopulmonary resuscitation
ROSC	Return of spontaneous circulation
ACLS	Advanced cardiovascular life support
ICU	Intensive care unit
BiPAP	Bilevel positive airway pressure
CK	Creatine kinase
ECG	Electrocardiography
